# Contribution of *α*‐synuclein cytopathologies to distinct seeding of misfolded *α*‐synuclein

**DOI:** 10.1111/bpa.70024

**Published:** 2025-06-16

**Authors:** Ain Kim, Ivan Martinez‐Valbuena, Krisztina Danics, Shelley L. Forrest, Gabor G. Kovacs

**Affiliations:** ^1^ Department of Laboratory Medicine & Pathobiology University of Toronto Toronto Ontario Canada; ^2^ Tanz Centre for Research in Neurodegenerative Disease University of Toronto Toronto Ontario Canada; ^3^ Krembil Brain Institute, University Health Network Toronto Ontario Canada; ^4^ Department of Forensic and Insurance Medicine Semmelweis University Budapest Hungary; ^5^ Neuropathology and Prion Disease Reference Center, Department of Forensic and Insurance Medicine Semmelweis University Budapest Hungary; ^6^ Edmond J. Safra Program in Parkinson's Disease and the Morton and Gloria Shulman Movement Disorders Clinic Toronto Western Hospital Toronto Ontario Canada; ^7^ Laboratory Medicine Program University Health Network Toronto Ontario Canada

**Keywords:** alpha‐synuclein, cell‐specific, machine learning, seeding behavior

## Abstract

Synucleinopathies are a group of neurodegenerative diseases characterized by the deposition of misfolded *α*‐synuclein (*α*Syn), predominantly in oligodendrocytes in multiple system atrophy (MSA) and in neurons in Lewy body diseases (LBD). The contribution of *α*Syn cytopathologies to the pathogenesis of these diseases is underappreciated. Seed amplification assays of MSA and LBD brains have revealed striking differences in *α*Syn seeding between regions and cases. Therefore, our aim was to evaluate whether different brain regions containing distinct *α*Syn cytopathologies contribute to different seeding characteristics. We collected 2‐mm micro‐punches of regions in MSA (*n* = 10) and LBD (*n* = 15) cases from formalin‐fixed paraffin‐embedded tissues. We performed double immuno‐labeling for disease‐associated *α*Syn and cellular markers on tissue microarrays, evaluated co‐deposition of other neurodegenerative disease‐related proteins and, from the same micro‐punched samples, we analyzed *α*Syn seeding. Based on these variables, machine learning algorithms were used to reduce dimensionality of the dataset and cluster the regions in MSA and LBD cases, revealing that different compositions of *α*Syn cytopathologies influence *α*Syn seeding patterns. Our results support the notion of different cellular processing of *α*Syn and its contribution to the variability in seeding. This has implications for understanding disease progression, interpretation of seed amplification assays, and opens avenues for the development of cell type‐specific antibodies against *α*Syn.

## Graphical abstract

This study investigated whether brain regions with distinct predominance of *α*‐synuclein (*α*Syn) cytopathologies show different *α*Syn seeding patterns in multiple system atrophy (MSA) and Lewy body disease (LBD), using an interdisciplinary approach. High seeding activity is observed in regions with oligodendrocytic‐predominant αSyn pathology in MSA. In regions with combined neuronal cytoplasmic‐ and astrocytic‐αSyn pathology, the increased involvement of astrocytes shows higher seeding activity in LBD. Lower seeding activity is observed in regions with predominant neuronal cytoplasmic‐*α*Syn in LBD.

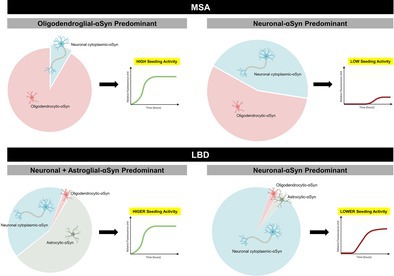

## 1 INTRODUCTION

Synucleinopathies belong to a group of neurodegenerative diseases that are pathologically characterized by the loss of neurons and deposition of misfolded *α*‐synuclein (*α*Syn). Clinically, these comprise Parkinson's disease (PD), Parkinson's disease with dementia (PDD), and dementia with Lewy bodies (DLB) grouped neuropathologically as Lewy body diseases (LBD), and multiple system atrophy (MSA). LBD is characterized by αSyn inclusions in neurons, in the form of Lewy bodies and Lewy neurites, while MSA is characterized by the deposition of *α*Syn in oligodendrocytes, termed glial cytoplasmic inclusions (GCIs) or Papp‐Lantos bodies [1].

MSA is usually referred to as oligodendrocytic‐predominant synucleinopathy and LBD as neuronal‐predominant synucleinopathy, underappreciating the potential role of other cytopathologies in the pathobiology [2, 3, 4]. However, it is well established that depending on antibodies targeting different epitopes and the stage of sequential anatomical involvement, some brain regions also show astrocytic and oligodendrocytic *α*Syn inclusions in LBD and neuronal αSyn inclusions in MSA [5, 6, 7, 8, 9, 10]. Specifically, astrocytic *α*Syn immunoreactivity in LBD is frequently observed in Braak stages 4 and above, mainly in the striatum and medial temporal lobe structures [7, 8, 11, 12]. Astrocytic *α*Syn inclusions show star‐like morphologies in the cortex and amygdala, while perinuclear granular deposits of *α*Syn are observed mostly in the astrocytes of the striatum [11]. Additionally, oligodendrocytic αSyn inclusions are consistently observed in LBD cases with Braak Stage 3 and above in the substantia nigra and thalamus (i.e., the pallidothalamic tract) [8, 13, 14]. On the other hand, neuronal *α*Syn inclusions in MSA are consistently observed in cortical areas, hippocampus, striatum, thalamus, subthalamus, locus coeruleus, pontine base, inferior olives, substantia nigra, or cerebellum [9, 15, 16].

Distinct biochemical and molecular characteristics of MSA and LBD are driven, at least in part by the different structures of the misfolded *α*Syn filaments seen under cryogenic‐electron microscopy (cryo‐EM) and represented by different seeding kinetics of misfolded αSyn in the brain tissue and cerebrospinal fluid using seed amplification assays (SAA), specifically the real‐time quaking‐induced conversion (RT‐QuIC) assay [17, 18, 19, 20, 21]. SAA is a robust technique that utilizes the self‐propagating properties of misfolded forms of *α*Syn to amplify and detect minute amounts of *α*Syn aggregates. The cycles of shaking and incubation promote fragmentation and elongation of the *α*Syn seeds, and with each cycle, the number of active seeds increases, promoting the recruitment of substrates for conversion and elongation [22, 23, 24]. The amplified *α*Syn aggregates can be detected real‐time using a fluorescence molecule called thioflavin T (ThT) which binds to *β*‐sheet structures of the pathological *α*Syn [25]. However, one caveat is that SAAs are typically performed using proteins extracted from frozen or fresh tissue, lacking cytopathological context. It remains challenging to locate the precise area of the sample that contains cell type‐specific *α*Syn at the time of collection, without immunohistochemical confirmation. Performing immunohistochemistry (IHC) on the frozen sample prior to the SAA is possible; however, the quality of morphology is not comparable to the morphologies evaluated using fixed tissue. Even methods that attempt to evaluate cytopathology on one side of the brain using fixed tissue and evaluate seeding on the contralateral sides of the brain are limited by spatial and hemispheric variability since a millimeter difference in sampling location can result in significant differences in the composition of dominant cell type‐specific *α*Syn. Therefore, we evaluated seeding of *α*Syn in the same sample where IHC demonstrated *α*Syn cytopathology. To achieve our goal, we used an established protein extraction method for formalin‐fixed paraffin‐embedded (FFPE) human brain tissue, which has been demonstrated to be reliable for *α*Syn seeding [26, 27].

Recent studies from our group have demonstrated seeding differences between cases with the same disease (i.e., low‐ and high‐seeders with different area under the curve) and also between brain regions of MSA and LBD [27, 28]. Given that these brain regions have different cell type‐specific accumulation of *α*Syn (i.e., neuronal, astrocytic, or oligodendrocytic), here we investigate whether brain regions with distinct predominance of *α*Syn cytopathologies influence *α*Syn seeding patterns. To overcome challenges in analyzing complex datasets containing numerous features, machine learning, which is essentially a set of mathematical algorithms allowing unbiased analysis of the multi‐dimensional data, was used to unravel data patterns that are not obvious to the researcher.

## 2 MATERIALS AND METHODS

A summary of the methods is schematically outlined in online Supplementary Figure 1.

### 2.1 Case selection

Ten cases with MSA (5 females) and fifteen cases with LBD (7 females) were selected from the University Health Network‐Neurodegenerative Brain Collection (UHN‐NBC, Toronto, ON, Canada) and the Neuropathology Archives of the National Institute of Psychiatry and Neurology (Semmelweis University, Budapest, Hungary; GGK and KD). Five cases lacking *α*Syn pathology (2 females) were also selected as control samples for the SAA. Autopsied human brain tissue was collected with informed consent from patients or their next of kin with the approval of local institutional review boards. All cases were systematically examined for co‐pathologies [29, 30, 31, 32, 33, 34, 35, 36, 37, 38]. Demographics and neuropathological evaluations are summarized in Table 1. To control for potential chemical modifications that may affect seeding differences, all FFPE tissues in this study have been fixed in 10% neutral buffered formalin for 2 weeks.

**TABLE 1:** Demographic and neuropathological diagnosis of cases used in the study. Controls represent cases without IHC‐detected *αSyn* pathology.

Case	Sex	Age at death	PMD (h)	Clinical subtype	Lewy Braak stage	NFT Braak stage^a^	Thal phase^a^	ADNC^a^	LATE‐NC stage^a^	Other neuropathological diagnosis
LBD1	M	80	6	DLB	5	4	3	Intermediate	0	None
LBD2	M	76	3.5	DLB	4	4	3	Intermediate	0	CAA^a^ (type 2), hypertensive vessel disease
LBD3	F	78	59	DLB	4	4	3	Intermediate	0	ARTAG^a^ Amygdala, AGD^a^ (Stage II), CAA (type 2), Chronic adhesive spinal arachnoiditis
LBD4	M	67	9	DLB	5	4	4	Intermediate	0	Lacunar infarct
LBD5	F	83	4.5	DLB	5	5	4	High	2	None
LBD6	F	92	6	DLB	5	3	3	Intermediate	0	None
LBD7	M	75	47	PD	5	2	1	Low	0	ARTAG Hippocampus and Amygdala, PART^a^ (possible)
LBD8	F	73	4.5	DLB	5	4	4	Intermediate	0	CAA (type 2)
LBD9	F	94	70	DLB	4	3	0	Not AD	0	ARTAG Amygdala, PART (Braak stage III)
LBD10	M	83	56	PDD	4	2	2	Low	0	None
LBD11	M	62	120	PDD	4	2	0	Not AD	0	PART (Braak stage II), AGD (stage I), CAA (type 2)
LBD12	F	71	N/A	PD	5	3	3	Intermediate	0	CAA (type 1)
LBD13	M	79	35	PD	4	2	1	Low	0	AGD (Stage III), CAA (type 2), Lacunar state in Basal Ganglia and Thalamus
LBD14	M	69	N/A	PD‐MCI	5	2	3	Low	2	ARTAG Medial Temporal Lobe, CAA (type 2)
LBD15	F	74	48	PD^b^	4	2	0	Not AD	0	PART (Braak stage II), CAA (type 2), Arteriolosclerosis
MSA1	M	64	24	MSA‐C	N/A	2	0	Not AD	0	PART (Braak stage II), AGD (Stage II)
MSA2	M	62	20	MSA‐C	N/A	2	0	Not AD	0	PART (Braak stage II), ARTAG Medial Temporal Lobe
MSA3	F	64	24	MSA‐P	N/A	2	5	Low	0	CAA (type 1)
MSA4	F	61	45	MSA‐P	N/A	1	0	Not AD	0	PART (Braak stage I)
MSA5	F	78	18	MSA‐P	N/A	2	2	Low	0	AGD (Stage II), CAA (type 2)
MSA6	M	66	24	MSA‐P	N/A	1	1	Low	0	CAA (type 2)
MSA7	M	69	24	MSA‐C	N/A	1	3	Low	0	Age‐related limbic p62 neuritic profiles
MSA8	F	61	N/A	MSA‐C	N/A	2	4	Low	0	CAA (type 1)
MSA9	F	66	18	MSA‐C	N/A	1	1	Low	0	Age‐related limbic p62 neuritic profiles
MSA10	M	43	18	MSA‐C	N/A	0	0	Not AD	0	None
Control1	F	52	28	N/A	N/A	0	0	Not AD	0	None
Control2	M	24	48	N/A	N/A	0	0	Not AD	0	None
Control3	M	20	N/A	N/A	N/A	0	0	Not AD	0	None
Control4	F	64	24	N/A	N/A	0	0	Not AD	0	Microinfarct in Globus Pallidus
Control5	M	64	N/A	N/A	N/A	2	0	Not AD	0	PART (Braak stage II), Metastasis in Thalamus

Abbreviations: AD, Alzheimer's disease; ADNC, Alzheimer's disease neuropathologic change; AGD, argyrophilic grain disease; ARTAG, aging‐related tau astrogliopathy; CAA, cerebral amyloid angiopathy; DLB, dementia with Lewy bodies; IHC, immunohistochemistry; LATE‐NC, limbic‐predominant age‐related TDP‐43 encephalopathy neuropathologic change; LBD, Lewy body disease; MSA, multiple system atrophy; MSA‐C, multiple system atrophy‐cerebellar type; MSA‐P, multiple system atrophy‐parkinsonian type; N/A, not available or not applicable; NFT, neurofibrillary tangle; PART, primary age‐related tauopathy; PD, Parkinson's disease; PDD, Parkinson's disease with dementia; PD‐MCI, Parkinson's disease with mild cognitive impairment; PMD, post‐mortem delay.

^a^ The level of ADNC was evaluated using the Braak neurofibrillary tangle (NFT) stage [30], Thal phase [31], and the Consortium to Establish a Registry for Alzheimer's Disease (CERAD) criteria for amyloid‐β deposition [32]; TDP‐43 pathology characterized according to LATE‐NC [33], AGD classified based on Saito stage [34], ARTAG [35] and PART [36] according to the harmonized evaluation strategies, and CAA based on the type 1 and type 2 neuropathological criteria [37].

^b^ Clinically atypical parkinsonism including the suspicion of MSA‐P but neuropathology shows unequivocal pathology of LBD.

### 2.2 Tissue microarray (TMA)

For each MSA case, disease‐associated *α*Syn (5G4)‐immunostained sections were evaluated under the microscope to delineate areas with predominant *α*Syn cytopathology: *α*Syn neuronal cytoplasmic inclusions (NCIs) in the locus coeruleus and inferior olivary nucleus, and oligodendrocytic‐*α*Syn inclusions in the cerebellar white matter (WM). For LBD cases, neuronal *α*Syn pathology (summarized as NCIs) was defined as typical brainstem or cortical type Lewy bodies or fine dot‐like or small compact NCIs, which were located in the temporal cortex, locus coeruleus, and substantia nigra; astrocytic‐*α*Syn inclusions in the temporal cortex, putamen, hippocampus, and amygdala; and oligodendrocytic‐*α*Syn inclusions in a circumscribed area of the pallidothalamic tract. The delineated slides were then placed and aligned on top of the corresponding FFPE tissue block that had been melted at 60°C for 20 min, and a clean 2‐mm micro‐punch was used to collect the tissue area for each tissue block. The micro‐punched cores were fitted into the tissue microarray (TMA) mold, and sections were cut with the microtome for confirmation by IHC.

### 2.3 IHC for *α*Syn and evaluation of co‐pathology

Each slide containing the TMA cores was stained with the anti‐*α*Syn (5G4; 1:4000; 5 min pre‐treatment with 80% formic acid; Roboscreen, Leipzig, Germany). A semi‐quantitative score (0: none, 1: mild, 2: moderate, 3: severe, 4: extremely severe) of each αSyn cytopathology (i.e., neuronal cytoplasmic‐, neuritic‐ astrocytic‐, and oligodendrocytic‐*α*Syn) was provided for each TMA core by a neuropathologist (GGK). This was used to validate the image analysis results. For co‐pathology, semi‐quantitative scores (0: none, 1: mild, 2: moderate, 3: severe) were used to evaluate the severity of phosphorylated‐tau (AT8; 1:1000; Thermo Fisher Scientific, MA, USA) immunoreactive neurofibrillary tangles and amyloid‐*β* (6F/3D; 1:50; 60 min pre‐treatment with 80% formic acid; Dako, Glostrup, Denmark) immunoreactive plaques (online Supplementary Figure 2).

### 2.4 Quantification of cell type‐specific *α*Syn

Proceeding deparaffinization and antigen retrieval, the TMA sections were double‐labeled with the following primary antibody—Alexa Fluor conjugated secondary antibody combinations:

- Anti‐*α*Syn (5G4; 1:1000; 5 min pre‐treatment with 80% formic acid; Roboscreen, Leipzig, Germany) labeled by Alexa Fluor‐488 (1:500; goat anti‐mouse; Invitrogen, MA, USA) combined with rabbit polyclonal anti‐MAP2 (1:500; ABclonal Technology, MA, USA) labeled by Alexa Fluor‐555 (1:500; donkey anti‐rabbit; Invitrogen, MA, USA) to identify neurons.
- Anti‐*α*Syn (5G4; 1:1000; 5 min pre‐treatment with 80% formic acid; Roboscreen, Leipzig, Germany) labeled by Alexa Fluor‐488 (1:500; goat anti‐mouse; Invitrogen, MA, USA) combined with guinea pig polyclonal anti‐GLT‐1 (1:1000, EMD Millipore Corp, MA, USA) labeled by Alexa Fluor‐555 (1:500; donkey anti‐guinea pig; Invitrogen, MA, USA). GLT‐1 was used as an astrocytic marker, since it also labels the distal astrocytic processes [39].
- Anti‐*α*Syn (5G4; 1:1000; 5 min pre‐treatment with 80% formic acid; Roboscreen, Leipzig, Germany) labeled by Alexa Fluor‐488 (1:500; goat anti‐mouse; Invitrogen, MA, USA) combined with rabbit polyclonal anti‐TPPP (TPPP/p25; 1:1000; [40]) labeled by Alexa Fluor‐555 (1:500; donkey anti‐rabbit; Invitrogen, MA, USA) to identify oligodendrocytes.

Each TMA slide was then stained with ProLong™ Gold antifade reagent with DAPI staining (Invitrogen, MA, USA). Double‐labeled immunofluorescence (IF) imaging was performed using a Nikon C2Si + confocal on a Nikon Ti2‐E inverted microscope. Images of each TMA core were captured using the Scan Large Image acquisition function on NIS‐Elements (v5.30.04).

### 2.5 Image analysis

For quantification of *α*Syn‐cell marker co‐localization, the object colocalization module in the image analysis software, HALO (Indica Labs, NM, USA), was used. Images of each TMA core were cropped to the same size and the total percentage of the co‐localized objects in each TMA circle was determined as the following:

$$\%\text{Colocalized Cells}=\frac{\#\text{of}\;5G4\;\text{with Cell Markers}}{\#\text{of Cell Marker Positive Cells}}$$

Due to the difficulty in isolating neuritic‐*α*Syn (i.e., Lewy neurites) in each image, the semi‐quantitative scores from IHC confirmation (GGK) were used in the machine learning analysis. In addition, the overlapping processes of GLT‐1‐positive cells were difficult to detect as one entity; therefore, manual counting was performed according to the rule outlined in online Supplementary Figure 3.

### 2.6 Protein extraction and quantification

The cores were carefully removed from the TMA, and proteins were extracted using the previously established protocol [41]. Thus, we used the same sample for SAA as for IHC. Following protein extraction, the bicinchoninic acid protein (BCA) assay (Thermo Scientific, MA, USA) was performed to determine the concentration of total protein in each sample, suspended in 1X PBS with protease inhibitor. The BCA standards were also prepared with 1X PBS with protease inhibitor.

### 2.7 αSyn SAA

SAA was performed as previously described, with a slight variation [27, 28, 41]. Importantly, this method was chosen based on the confirmation that disease‐specific seeding patterns detected in the gold‐standard SAA using frozen tissue were preserved using fixed brain tissue. Briefly, the SAA reaction mixture was composed of 40 mM phosphate buffer (pH 8.0), 350 mM Na_3_Citrate (Sigma, MI, USA), 0.1 mg/mL K23Q mutant sequence human recombinant *α*Syn (Impact Biologicals, PA, USA) and 10 μM ThT (Sigma, MI, USA). Recombinant *α*Syn was thawed and filtered, and 1 μg of PBS‐solubilized total protein extracted from the FFPE micro‐punch was added to the reaction mixture. To confirm the validity of each SAA and control for false positive reactions such as spontaneous aggregation, every plate included one non‐diseased positive control (pre‐formed *α*Syn fibrils), one disease‐confirmed positive control (protein extracted from whole FFPE tissue block of MSA or LBD), and six negative controls (five cases lacking *α*Syn pathology and one with deionized water). The plate was sealed and placed in the BMG FLUOstar Omega plate reader, where both the MSA‐favoring protocol and LBD‐favoring protocol were used as previously described [27, 28, 41]. The MSA‐favoring protocol amplifies MSA seeds more than LBD seeds, while the LBD‐favoring protocol amplifies LBD seeds more than those of MSA. Each protocol allows the comparison between SAA parameters across the different cases of the corresponding disease. All MSA curves in this study are reported using the MSA‐favoring protocol, while all LBD curves are reported using the LBD‐favoring protocol. The threshold was calculated as the averaged relative fluorescence unit (%RFU) of the baseline fluorescence plus 3 standard deviations, and the cut‐off time was determined as 20 h for both MSA‐ and LBD‐favoring protocols, based on the earliest time that the negative controls crossed the threshold. Four replicates of each sample were evaluated. All data was normalized, and kinetic parameters were determined by fitting the four‐parameter logistic model to the averaged fluorescence data using Python (version 3.10). The lag phase, T50, and maximum ThT level are used to describe differences in seeding activity, while “low”‐ and “high‐seeding” are defined by the area under the curve.

### 2.8 Sandwich‐ELISA

LEGEND MAX™ Human *α*Syn (Colorimetric) ELISA kits (San Diego, California, USA) were used to measure total αSyn in each protein sample, according to the manufacturer's protocol and as previously described [6].

### 2.9 Statistical analysis

All data were analyzed and plotted using Prism (v9, GraphPad Software, San Diego, CA). All bar graphs represent mean ± SEM. Comparisons were made using the two‐tailed Mann–Whitney U test for comparing two groups, and the Kruskal–Wallis test with Dunn's multiple comparisons test was used for comparing three or more groups. For each Friedman test with Dunn's multiple comparisons test that was used for comparing overall differences in three or more groups with paired data, Wilcoxon pairwise‐comparison was also evaluated. Non‐parametric Spearman correlation was used for correlation analysis, with *p*‐values less than 0.05 regarded as significant. Principal component analysis (PCA) was performed on the normalized dataset to reduce dimensionality, followed by k‐means clustering performed on the PCA results. Quantified pathological features (i.e., kinetic parameters, proportions of cell type‐specific *α*Syn and co‐deposition of tau and amyloid‐*β*) were used in the analysis. Demographic and other neuropathological variables (i.e., case number, brain region, post‐mortem delay, age at death, Braak stages of Lewy‐related pathology, Alzheimer's disease neuropathologic change, clinical subtype and biological sex) were not included in the PCA and k‐means clustering but were compared in post‐hoc analysis. The pairwise plot, together with the elbow method, explained variance, distance from centroid, and silhouette score was used to determine the optimal principal component and number of clusters. Both k‐means clustering and PCA were performed in Python using the publicly available scikit‐learn library (https://scikit‐learn.org/stable/modules/clustering.html#clustering).

## 3 RESULTS

### 3.1 *α*Syn‐immunoreactive cytopathologies

**FIGURE 1:**
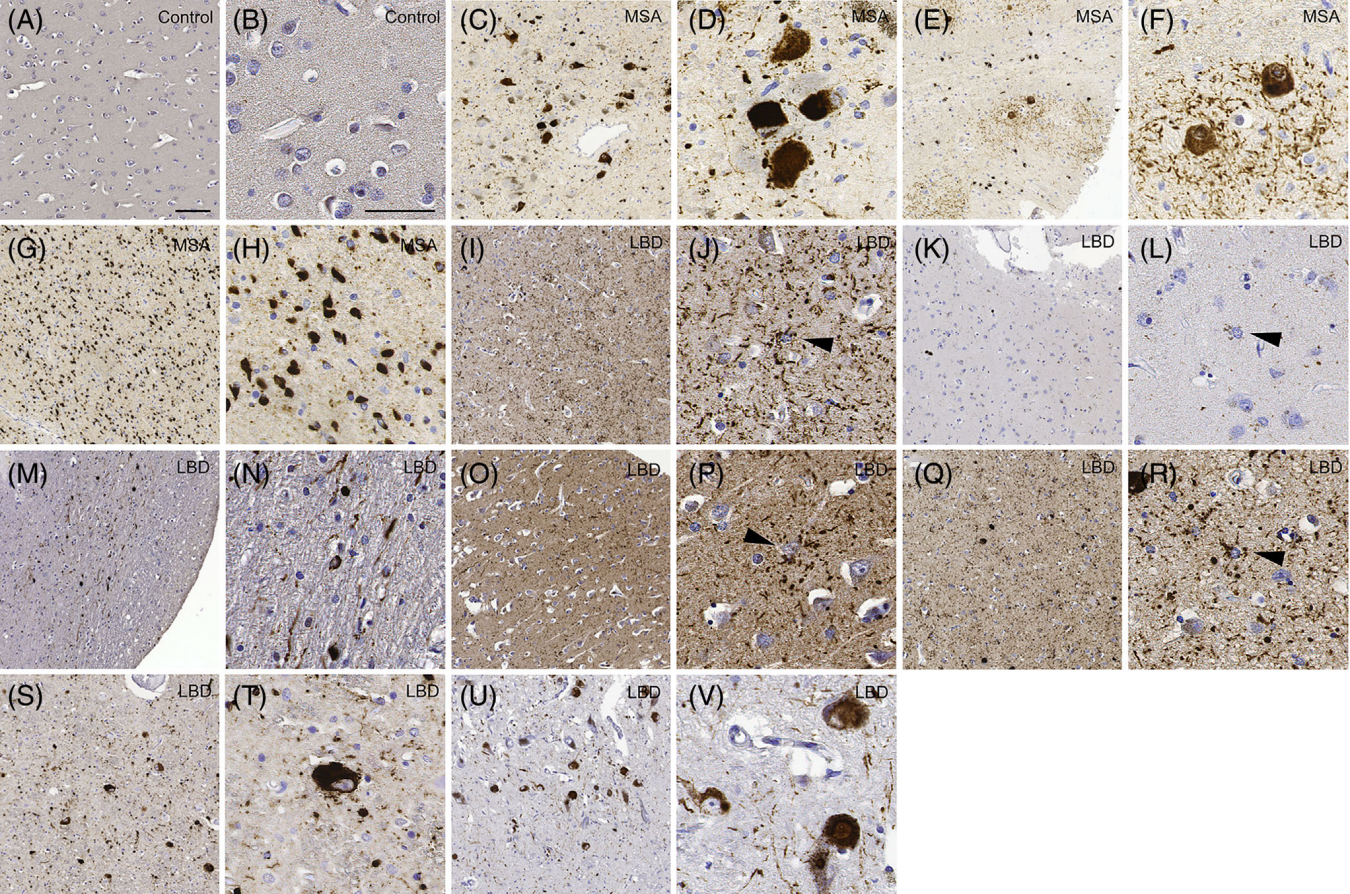
Confirmation of cell type‐specific 5G4‐immunoreactivy in different regions of MSA, LBD and neuropathological controls on TMA at low and high magnification. A representative case of the neuropathological controls with lack of *α*Syn immunoreactivity (A), (B), neuronal‐*α*Syn inclusions (i.e., neuronal cytoplasmic and neuritic) in the locus coeruleus of a representative MSA case (C), (D), neuronal‐*α*Syn inclusions with different morphology in the inferior olivary nucleus (E), (F), and oligodendrocytic‐*α*Syn inclusions in the cerebellar WM (G), (H). A representative case of LBD cohort with astrocytic *α*Syn in the temporal cortex with an arrowhead indicating star‐like morphology (I), (J) and the putamen with an arrowhead indicating dot‐like morphology (K), (L), oligodendrocytic *α*Syn in the pallidothalamic tract (M), (N), astrocytic *α*Syn with dot‐like morphology indicated by an arrowhead in the hippocampus (O), (P), star‐like astrocytic *α*Syn indicated by an arrowhead in the amygdala (Q), (R), neuronal‐*α*Syn inclusions in the locus coeruleus (S), (T) and the substantia nigra (U), (V) are shown. Scale bar in (A) represents 50 μm and applies to (C, E, G, I, K, M, O, Q, S, U) (low magnification images) and scale bar in (B) also represents 50 μm and applies to (D, F, H, J, L, N, P, R, T, V) (high magnification images). LBD, Lewy body disease; MSA, multiple system atrophy; TMA, tissue micro array; WM, white matter.

Microscopic evaluation of the TMA cores immunostained with the 5G4 antibody confirmed the precise collection of samples with distinct predominance of *α*Syn cytopathologies or the absence of immunoreactivity in cases lacking αSyn pathology (Figure 1, A, B). Consistent with the literature, NCIs in all MSA cases were found in the locus coeruleus with a skein‐like morphology or a dense inclusion that covered the whole cytoplasm while NCIs in the inferior olivary nucleus of the medulla oblongata had a cloud‐like morphology surrounding the dense inclusion [9, 16], and the oligodendrocytic inclusions in the cerebellar WM had a cone‐ or globular‐shaped morphology (Figure 1, C–H). In LBD (Figure 1, I–V), brainstem‐type Lewy bodies and other NCIs were identified in the locus coeruleus and substantia nigra, and cortical‐type Lewy bodies and other NCIs were identified in the temporal cortex and amygdala. The oligodendrocytic *α*Syn inclusions in the pallidothalamic tract showed coiled‐body or globular morphology. Lastly, the astrocytic *α*Syn inclusions were mostly star‐like in the temporal cortex and amygdala, while the putamen and hippocampus showed more dot‐like morphology surrounding the nucleus. All LBD cases examined in this study had oligodendrocytic‐ and astrocytic‐*α*Syn immunoreactivity.

### 3.2 Proportion of *α*Syn cytopathologies in each TMA

**FIGURE 2:**
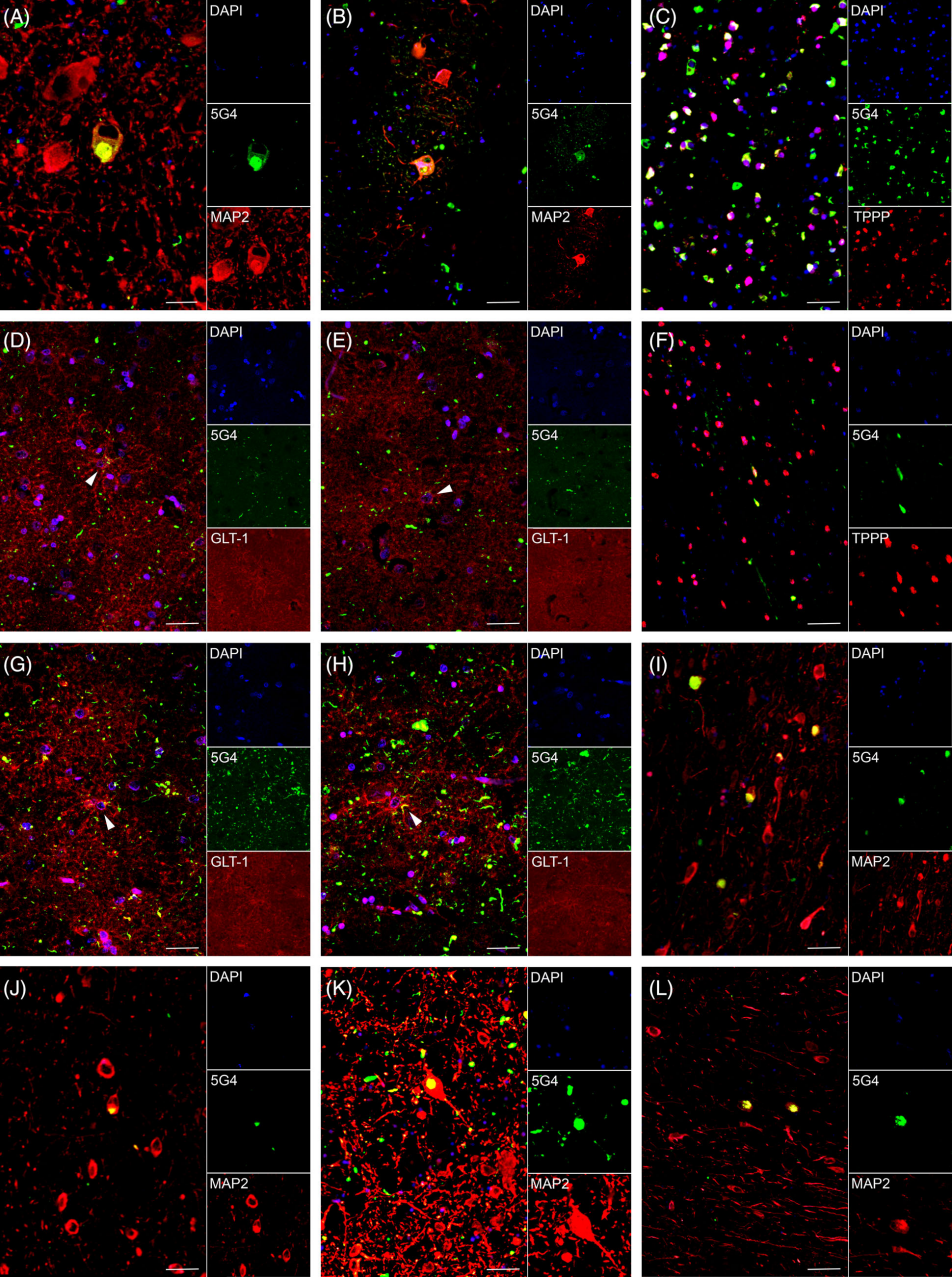
Co‐localization of 5G4 and cell marker in MSA and LBD. In MSA, the neuronal cytoplasmic‐*α*Syn inclusions in the locus coeruleus (A) and inferior olivary nucleus (B) are indicated by co‐localized 5G4 and MAP2, while the oligodendrocytic‐αSyn in inclusions in the cerebellar WM are indicated by co‐localized 5G4 and TPPP (C). In LBD, the astrocytic‐*α*Syn with different morphologies in the temporal cortex (D) and putamen (E) are marked by co‐localized 5G4 and GLT‐1 (indicated by the white arrowheads), the oligodendrocytic‐*α*Syn in the pallidothalamic tract (F) are indicated by co‐localized 5G4 and TPPP, different morphologies of astrocytic‐*α*Syn in the hippocampus (G) and amygdala (H) are marked by co‐localized 5G4 and GLT‐1 (indicated by the white arrowheads), while neuronal cytoplasmic‐αSyn inclusions in the hippocampus (I), amygdala (J), locus coeruleus (K), and substantia nigra (L) are indicated by co‐localized 5G4 and MAP2. 5G4‐immunoreactivity is labeled in green, cell markers are labeled in red and DAPI nucleus staining is labeled in blue. Scale bars represent 50 μm. LBD, Lewy body disease; MSA, multiple system atrophy; WM, white matter.

**FIGURE 3:**
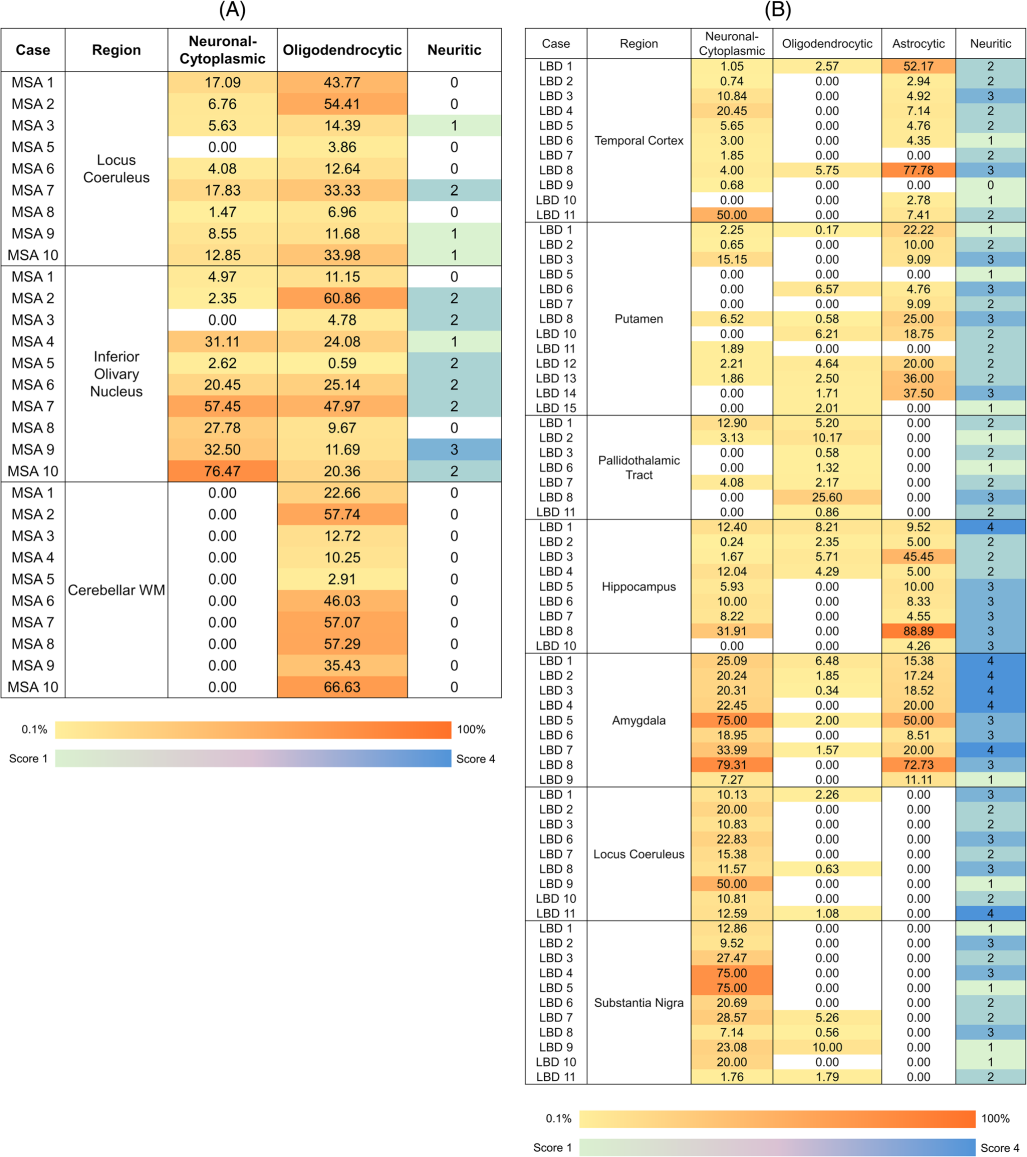
Proportion of co‐localized 5G4 and cell markers in each TMA core of MSA and LBD cases. The co‐localized 5G4 and cell markers serve as a measure of quantifying the proportion of cell type‐specific αSyn in each TMA core in MSA (A) and LBD cases (B). The values represent the number of 5G4‐positive cell‐types divided by the total number of cell‐types in each cropped TMA core of the same size, converted to %. For example, the higher the %, the higher the proportion of *α*Syn in the corresponding cell‐type. The color scale bar represents lowest to highest %, where yellow represents the lowest value and orange represents the highest value. The neuritic‐*α*Syn uses a different color scale bar, where green represents the lowest score and blue represents the highest score of neuritic‐*α*Syn severity, evaluated by a neuropathologist. 0 is represented by white. LBD, Lewy body disease; MSA, multiple system atrophy; TMA, tissue micro array; WM, white matter.

The cell type‐specific *α*Syn immunoreactivities were evaluated using double‐IF staining (Figure 2) and quantified by measuring the proportion of neuronal cytoplasmic‐ and oligodendrocytic‐*α*Syn for different regions of MSA (Figure 3A). Additionally, astrocytic‐*α*Syn was determined for different regions of LBD cases (Figure 3B). There was a wide heterogeneity in the predominance of cell type‐specific *α*Syn even in the same regions across different cases, and a wide range of *α*Syn cytopathology proportions in each region. This is not only due to the difference in the severity of *α*Syn pathology and the different cell types affected in each region, but also because of the method of sample collection. In some cases, the predominant *α*Syn cytopathology may not be accurately captured in the TMA core. For example, the inferior olivary nucleus of MSA 3 or the locus coeruleus of MSA 5 did not have any neuronal‐*α*Syn inclusion but only contained GCIs, indicating that the exact location delineated under the microscope was not accurately captured. This was likely due to the relatively small area of the inferior olivary nucleus or locus coeruleus available for the micro‐punch.

### 3.3 *α*Syn seeding in each region harboring different proportions of *α*Syn cytopathologies

**FIGURE 4:**
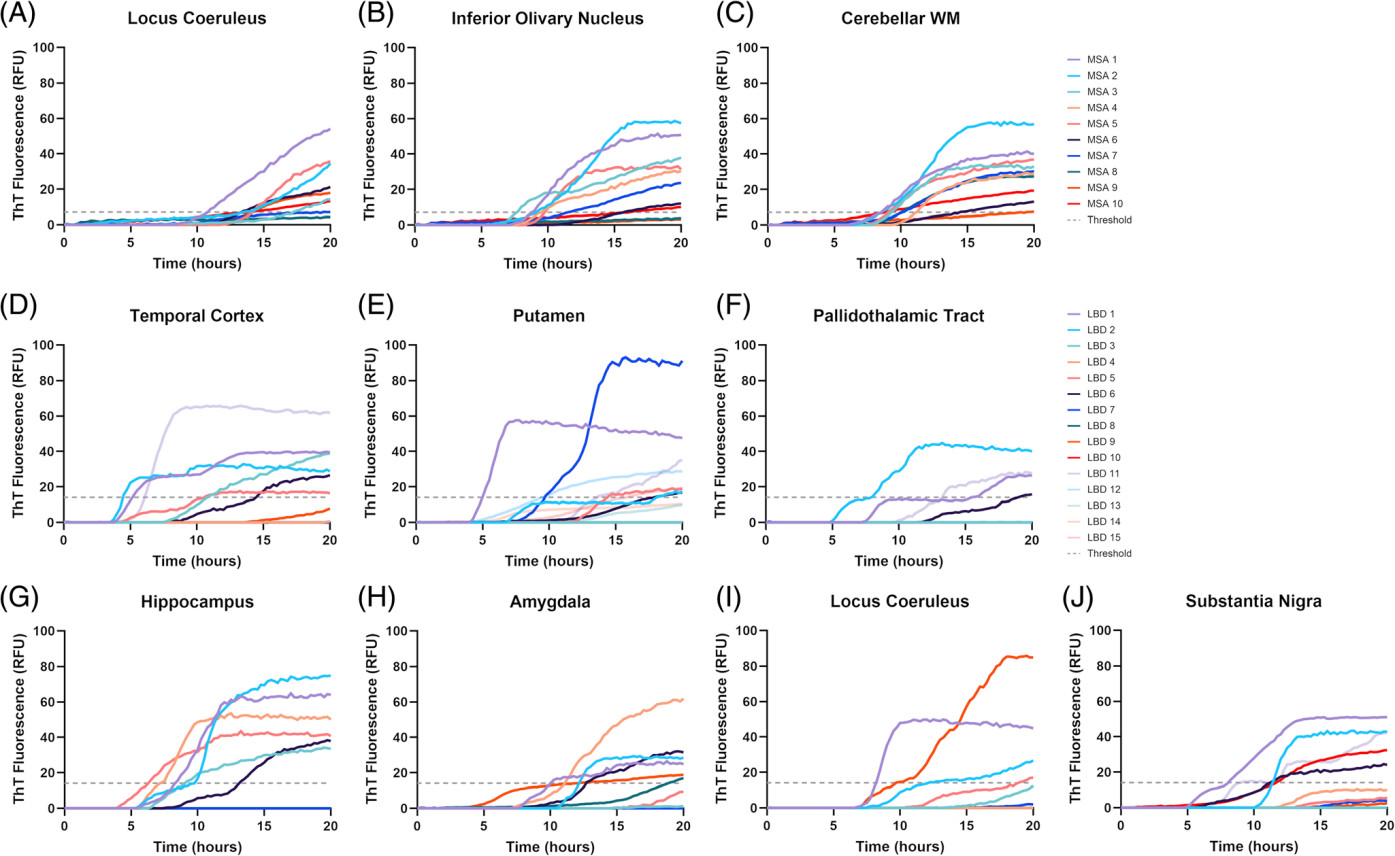
*α*Syn seeding in each TMA core taken from different regions of MSA and LBD cases. In MSA (*n* = 10), *α*Syn seeding activity was evaluated in the locus coeruleus (A), inferior olivary nucleus, (B) and the cerebellar WM (C). In LBD (*n* = 15), *α*Syn seeding activity was evaluated in the temporal cortex (D), putamen (E), pallidothalamic tract (F), hippocampus (G), amygdala (H), locus coeruleus (I) and substantia nigra (J). Those that did not cross the threshold before the cut‐off time were still included in the analysis. Each kinetic curve represents an average of 4 replicates; error bars have been removed for visualization purposes. Dashed line represents the threshold in MSA‐favoring protocol (A)–(C) and LBD‐favoring protocol (D)–(J). LBD, Lewy body disease; MSA, multiple system atrophy; RFU, relative fluorescence unit; ThT, thioflavin T; TMA, tissue micro array; WM, white matter.

We evaluated *α*Syn seeding in the same TMA cores that were used to confirm *α*Syn cytopathology in MSA (Figure 4, A–C) and LBD cases (Figure 4, D–J). As expected, the seeding behaviors of different proportions of cell type‐specific αSyn inclusions in each region were heterogeneous without any outstanding pattern. The control cases (*n* = 5) lacking αSyn pathology and the negative control (i.e., wells containing deionized water) did not show seeding within the cut‐off window. MSA and LBD samples that did not cross the threshold were still included in the analysis for the purpose of the study.

### 3.4 K‐means clustering on PCA results linking *α*Syn cytopathology and seeding

Due to the multi‐dimensional dataset consisting of several kinetic parameters (i.e., area under the curve, lag phase, T50 and maximum ThT), proportional *α*Syn cytopathology (i.e., varying % and combinations of neuronal cytoplasmic‐, neuritic‐. oligodendrocytic‐, and astrocytic‐*α*Syn) and co‐deposition of other misfolded proteins (i.e., tau and amyloid‐*β* pathology severity scores), PCA was used to reduce the dimensionality and identify the main sources of variation in the dataset. Further k‐means clustering of the PCA results can identify groups that share similar characteristics based on combined profiles, such as “weights” of cell type‐specific *α*Syn pathology and combinations of *α*Syn cytopathology associated with distinct seeding behavior.

**FIGURE 5:**
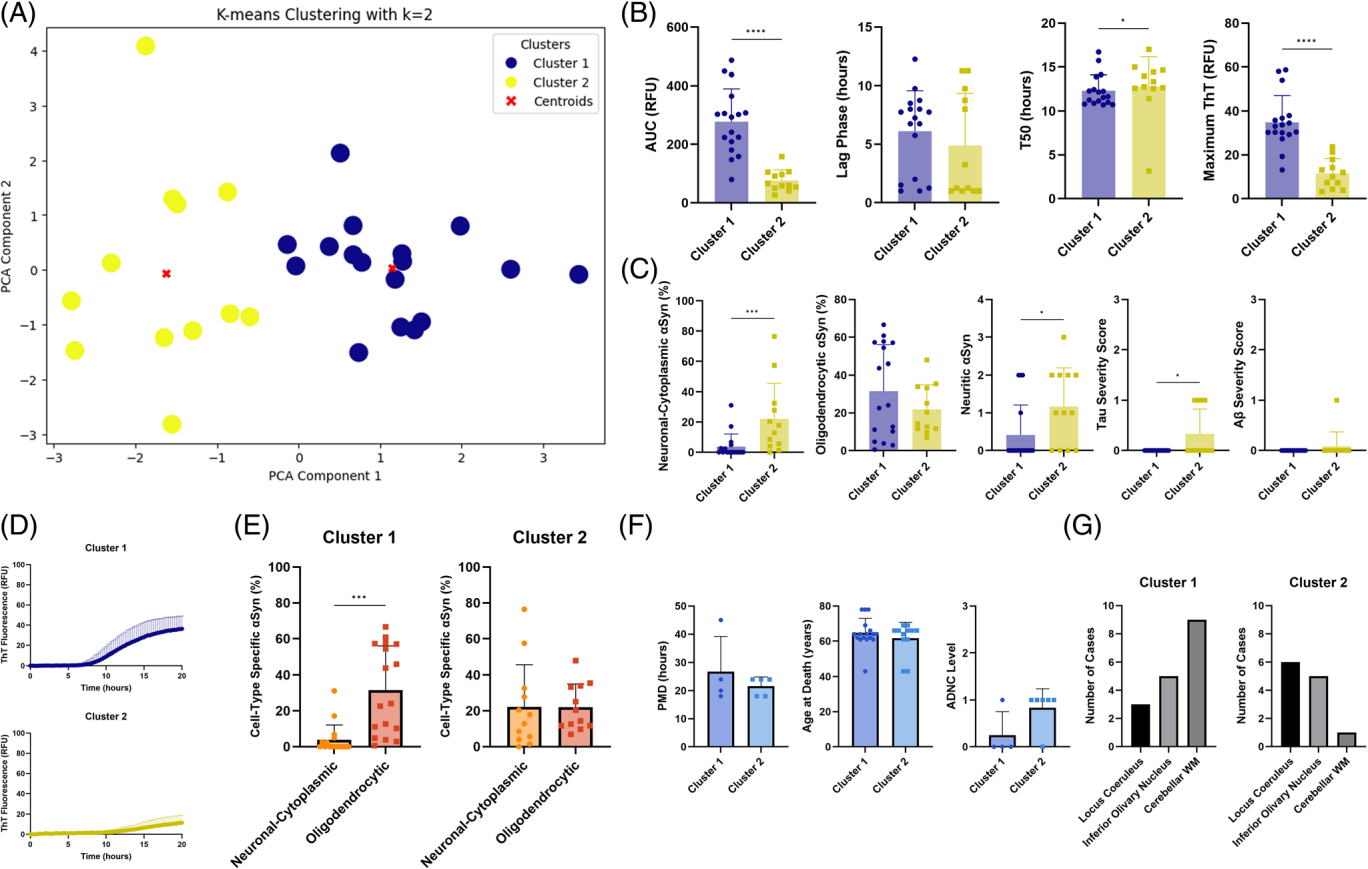
K‐means clusters of PCA results and post‐hoc comparisons in MSA. K‐means clustering of the first two principal components with 2 clusters visualized in a scatter plot, where blue represents cluster 1 and yellow represents cluster 2 (A). Post‐hoc comparisons of the features used in the machine learning analysis revealed significant differences in: (B) the kinetic parameters between clusters using Mann–Whitney U test, (C) cell type‐specific *α*Syn and tau and amyloid‐*β* severity scores between clusters using Mann–Whitney U test, (D) *α*Syn seeding activities between clusters, (E) proportion of *α*Syn cytopathology in each cluster using Wilcoxon signed‐rank test, and (F) demographical and other neuropathological features which did not have significant differences between clusters using Mann–Whitney U test. Demographical and other neuropathological features, indicated by blue bar graphs, have not been included in the dataset used for PCA and k‐means clustering, but were evaluated post‐hoc based on the clusters to which each data point was assigned. Evaluating the composition of brain regions in the clusters, cluster 1 consisted mostly of inferior olivary nucleus and cerebellar WM, while cluster 2 consisted of mostly the locus coeruleus, representing more involvement of the neuronal‐*α*Syn (G). *α*Syn, α‐synuclein; A*β*, amyloid‐*β*; ADNC, Alzheimer's disease neuropathologic change; AUC, area under the curve; PCA, principal component analysis; PMD, post‐mortem delay; RFU, relative fluorescence unit; ThT, thioflavin T.

Using the MSA dataset, the optimal number of components (i.e., each an “axis” representing the largest variation and the most important information in the data) was calculated to be 2 and the optimal number of clusters was determined as 2 (Figure 5A). With each datapoint assigned to a cluster based on the machine learning model, all features were re‐grouped into the clusters for post‐hoc analysis. We found that the brain regions grouped in cluster 1 had significantly higher area under the curve (*p* < 0.0001), shorter T50 (*p* = 0.0426), and higher maximum ThT (*p* < 0.0001) compared to those in cluster 2 (Figure 5B). Regarding *α*Syn cytopathology, cluster 2 had significantly higher % of neuronal cytoplasmic‐*α*Syn (*p* = 0.0008) and neuritic‐*α*Syn (*p* = 0.0353) compared to cluster 1 and for the presence of co‐pathology, cluster 2 had significantly higher tau severity score compared to cluster 1 (*p* = 0.0208; Figure 5C). Overall, cluster 1 had higher *α*Syn seeding activity compared to cluster 2 (Figure 5D). In addition to comparing the proportion of cell type‐specific *α*Syn between clusters, we evaluated the full profile of *α*Syn proportions for neuronal and oligodendrocytic cytopathologies within each cluster. We found that cluster 1, which had the highest seeding activity, consisted of regions with significantly more oligodendrocytic‐*α*Syn than neuronal cytoplasmic‐*α*Syn (*p* = 0.0003), confirming the oligodendrocytic‐dominant synucleinopathy of MSA (Figure 5E). Demographic and other neuropathological features were also evaluated post‐hoc based on the clusters to which each data point was assigned, and we found no significant differences of post‐mortem delay, age at death, Alzheimer's disease neuropathologic change, clinical subtypes (i.e., MSA‐cerebellar and MSA‐parkinsonian type; data not shown) and sex (data not shown) between the clusters (Figure 5F). When we examined the number of cases in each region of the different clusters in MSA, cluster 1 consisted mostly of the cerebellar WM and a few inferior olivary nucleus that harbors numerous GCIs, while cluster 2 consisted of mostly the locus coeruleus and a few inferior olivary nucleus, representing more involvement of the neuronal (i.e., neuronal cytoplasmic and neuritic) *α*Syn pathology (Figure 5G). Interestingly, one of the cerebellar WM that had strictly oligodendrocytic‐*α*Syn clustered together with samples with predominantly neuronal‐*α*Syn in cluster 2, due to similar “low” seeding behavior. Since our clustering approach integrates all variables, it is not unexpected that some samples with different cytopathologies could cluster together due to similarities in other variables.

**FIGURE 6:**
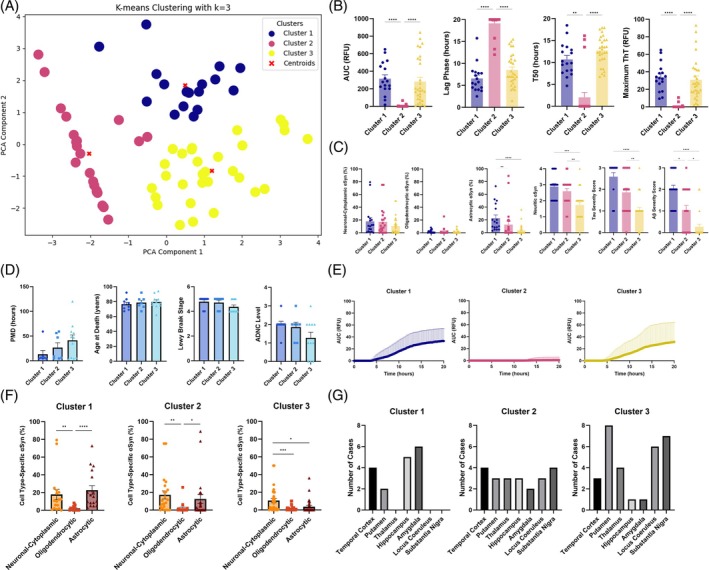
K‐means clusters of PCA results and post‐hoc comparisons in LBD. K‐means clustering of the first two principal components with 3 clusters visualized in a scatter plot, where blue represents cluster 1, pink represents cluster 2 and yellow represents cluster 3 (A). Post‐hoc comparisons of the features used in the machine learning analysis, where Kruskal–Wallis test revealed significant differences in: (B) the kinetic parameters between clusters, (C) cell type‐specific *α*Syn and tau and amyloid‐*β* severity scores between clusters, (D) demographical features which did not have significant differences between clusters, (E) *α*Syn seeding activities between clusters, and (F) proportion of *α*Syn cytopathology in each cluster using Friedman test corrected for multiple comparisons and Wilcoxon signed‐rank test. Demographical features, indicated by blue bar graphs, have not been included in the dataset used for PCA and k‐means clustering, but were evaluated post‐hoc based on the clusters to which each data point was assigned. Evaluating the composition of brain regions in the clusters, cluster 1 consisted of mostly the temporal cortex, hippocampus and amygdala which are affected in the later stages of disease, cluster 2 consisted of even distribution of all regions, and cluster 3 consisted of mostly the putamen, locus coeruleus and substantia nigra which are affected in the early stages of disease (G). *α*Syn, *α*‐synuclein; A*β*, amyloid‐*β*; ADNC, Alzheimer's disease neuropathologic change; AUC, area under the curve; PCA, principal component analysis; PMD, post‐mortem delay; RFU, relative fluorescence unit; ThT, thioflavin T.

Using the LBD dataset, the optimal number of components was calculated to be 2 and the optimal number of clusters was determined as 3 (Figure 6A). We found that the regions in cluster 1 had significantly higher area under the curve (*p* < 0.0001), shorter lag phase (*p* < 0.0001), higher T50 (*p* = 0.0017) and maximum ThT (*p* < 0.0001) compared to cluster 2, while cluster 3 also had significantly higher area under the curve (*p* < 0.0001), shorter lag phase (*p* < 0.0001), higher T50 (*p* < 0.0001) and maximum ThT (*p* < 0.0001) compared to cluster 2 (Figure 6B). Between cluster 1 and cluster 3, cluster 3 had longer lag phase and higher T50 compared to cluster 1, although not statistically significant but close to the conventional threshold (*p* = 0.0849 and *p* = 0.0641, respectively). Regarding *α*Syn cytopathology, cluster 1 had a significantly higher proportion of astrocytic‐*α*Syn than cluster 2 (*p* = 0.0071) and cluster 3 (*p* < 0.0001), while cluster 3 had a significantly lower proportion of neuritic‐*α*Syn than cluster 1 (*p* = 0.0002) and cluster 2 (*p* = 0.0020; Figure 6C). When we evaluated the co‐deposition of other misfolded proteins, we found that the tau severity score was significantly higher in cluster 1 compared to cluster 3 (*p* < 0.0001) and higher in cluster 2 compared to cluster 3 (*p* = 0.0087; Figure 6C). Amyloid‐*β* scores were significantly higher in cluster 1 compared to cluster 2 (*p* = 0.0180) and cluster 3 (*p* < 0.0001) while cluster 2 had significantly higher amyloid‐*β* scores compared to cluster 3 (*p* = 0.0156; Figure 6C). We found no significant difference in the post‐mortem delay, age at death, Braak stages of Lewy‐related pathology, Alzheimer's disease neuropathologic change, clinical subtypes (i.e., PD, PDD, DLB; data not shown) and sex (data not shown) between the clusters (Figure 6D). Overall, cluster 1 and cluster 3 had higher αSyn seeding activity compared to cluster 2 and cluster 3 had lower *α*Syn seeding activity compared to cluster 1 (Figure 6E). When we evaluated the profile or proportions of *α*Syn cytopathologies, we found that cluster 1 consisted of brain regions with significantly higher neuronal cytoplasmic‐*α*Syn (*p* = 0.0017) and astrocytic‐*α*Syn (*p* < 0.0001) compared to oligodendrocytic‐*α*Syn pathologies (Figure 6F). Cluster 2 also followed a similar trend, where the brain regions showed significantly higher neuronal cytoplasmic‐*α*Syn (*p* = 0.0014) and astrocytic‐*α*Syn (*p* = 0.0214) compared to oligodendrocytic‐*α*Syn pathology, but lower astrocytic‐*α*Syn compared to neuronal cytoplasmic‐*α*Syn pathology (Figure 6F). Further examining the cases in this cluster, we found that the k‐means clustering grouped regions with low or negative seeding, despite the presence of *α*Syn cytopathology, possibly representing a group of samples where the cell type‐specific contribution of seeding differences may not have been reflected. Finally, cluster 3 consisted of brain regions with significantly higher neuronal cytoplasmic‐*α*Syn compared to oligodendrocytic‐*α*Syn (*p* = 0.0005) and astrocytic‐*α*Syn pathology (*p* = 0.0187; Figure 6F). When we examined the number of cases in each region of the different clusters in LBD, cluster 1 consisted of mostly the temporal cortex, hippocampus and amygdala, which are affected in the later stages of disease. Cluster 2 had an even distribution of all regions with no single region emerging as prominent, and cluster 3 consisted of mostly the locus coeruleus and substantia nigra, which are affected in the early stages of disease, and the putamen (Figure 6G).

### 3.5 Correlation between variables and seeding kinetics

To investigate whether the seeding difference between the regions with distinct dominating *α*Syn‐cytopathologies was indeed due to the cytopathological differences and not the amount of *α*Syn seed in each sample, we performed sandwich‐ELISA and quantified the total *α*Syn concentration (pg/mL) in each protein sample that was used in the analysis. The amount of total *α*Syn per 1 μg of protein for each sample was calculated. We did not find any significant correlation between the amount of total *α*Syn and any of the kinetic parameters measured. However, a correlation existed between kinetic parameters where the area under the curve positively correlated with maximum ThT (*r* = 0.950, *p* < 0.0001) and negatively correlated with the lag phase (*r* = −0.692, *p* < 0.0001). Maximum ThT also negatively correlated with the lag phase (*r* = −0.591, *p* < 0.0001) and positively correlated with T50 (*r* = 0.250, *p* = 0.0131). The severity scores of tau and amyloid‐*β* did not significantly correlate with any of the SAA parameters.

## 4 DISCUSSION

Based on the distinct morphological and cell type‐specific accumulation of misfolded *α*Syn in synucleinopathies, our study attempted to zoom into the cellular level and link *α*Syn cytopathology with seeding differences using novel and interdisciplinary techniques (i.e., biochemistry, neuropathology and machine learning). We observed distinct *α*Syn seeding behaviors across different brain regions, each with a unique predominance of *α*Syn cytopathology.

The cytopathological make‐up in each brain region is not absolute, and αSyn cytopathologies can co‐exist even within a precise 2 mm‐diameter circle (Figure 1). Therefore, capturing *α*Syn inclusions with only one cell type with our micropunching method is extremely difficult, if not impossible. Collecting tissues from lower stages of disease would be ideal for locating areas with mostly one uniform cell type of *α*Syn inclusions, but these cases are rare, and as pathology has not fully developed, finding a sufficient number of *α*Syn‐positive cytopathologies would be challenging. The application of laser microdissection would also be challenging since double labeling would be required to identify *α*Syn cytopathologies, which, in frozen sections, would be impacted by reduced quality of morphology. Therefore, our study used a novel approach linking cytopathology on FFPE tissues with seeding, focusing on incorporating underappreciated *α*Syn cytopathologies that emerge in the later stages of disease. The advantage of our method is that we used the same samples for IHC and SAA. Indeed, in the same brain regions, we observed different seeding behaviors in both MSA and LBD (Figure 4). This raises a potential obstacle in interpreting the results as the features (i.e., the SAA parameters and proportion of cell type‐specific *α*Syn) are not independent of one another. Re‐grouping the samples based on the dominant *α*Syn cytopathology and comparing the seeding behaviors could provide insight; however, it may not be biologically relevant as it overlooks the contribution of less prominent *α*Syn cytopathologies present in the sample and their potential effect on the seeding behavior. Even correlation analysis of these features in all regions and all cases do not reveal patterns that are immediately obvious, as we assume the interactions to be non‐linear or multifactorial. To address these issues, we applied machine learning algorithms to capture the proportional aspects in each sample and its relation to the *α*Syn seeding kinetics in an unbiased manner.

In MSA, we observed a pattern confirming the predominance of oligodendrocytic‐*α*Syn pathology in MSA (summarized in Supplementary Figure 4). Since seeding is slower when there are higher proportions of neuronal‐*α*Syn pathology even in the presence of high proportions of oligodendrocytic‐*α*Syn, we speculate that the neuronal inclusions could be a permanent reservoir of *α*Syn seeds potentially reflecting the successful survival of the neurons after battling through the pathological *α*Syn aggregation process.

In LBD, the highest seeding activity was observed in two different scenarios, where: (i) there is a predominance of astrocytic‐*α*Syn pathology together with neuronal cytoplasmic‐*α*Syn pathology, and (ii) there is a predominance of neuronal cytoplasmic‐*α*Syn pathology without prominent glial *α*Syn pathology (summarized in Supplementary Figure 4). In the former scenario (scenario 1), regions that are affected in the later phases of Lewy Braak stage 5 were clustered together, possibly explaining the high accumulation of astrocytic‐*α*Syn and co‐deposition of other pathological proteins [42, 43]. However, the severity of tau and amyloid‐*β* pathology scores in each cluster did not significantly correlate with the seeding capacity, suggesting that the impact of co‐pathology on seeding may not be straightforward in this context. Although co‐pathology could play a significant role in neurodegeneration and disease progression, this is beyond the scope of our study. In the latter scenario (scenario 2), regions that are affected in the early phases of the disease clustered together, suggesting that, in contrast to MSA, these neuronal populations in LBD may harbor seeding‐prone *α*Syn species that can initiate pathological spread without astrocytic involvement, confirming the neuronal‐predominant nature of LBD. Comparing the scenarios, it is evident that with increasing astrocytic involvement in the presence of neuronal cytoplasmic‐*α*Syn, higher seeding activity is observed. Taken together, these observations suggest that *α*Syn aggregates accumulated in different cell types could be one of the factors that could contribute to different seeding behaviors, in addition to other unexplored factors.

The different *α*Syn seeding behaviors between the clusters and their dominating *α*Syn cytopathology points toward the potential role of distinct cellular milieus in the pathogenesis of *α*Syn aggregates [44, 45]. Peng et al. reported that the propagation of Lewy body‐type *α*Syn inside oligodendrocytes leads to the formation of GCI‐type strains, raising the hypothesis that cellular micro‐environment promotes the formation of distinct strains [45]. Previous studies have biochemically analyzed fractionated proteins from the pons of MSA cases that contain neuronal‐*α*Syn and identified carboxy‐truncated *α*Syn between residues 115–112 [16]. This suggests a different processing of *α*Syn by different cell types and that *α*Syn aggregates from neuronal populations could be released and internalized by oligodendrocytes, which also express *SNCA* transcripts, potentially leading to the conversion of *α*Syn into a more aggressive form in MSA [46]. In LBD, the cellular production and expression of *SNCA* transcripts in astrocytes are low‐level or absent compared to oligodendrocytes [47, 48, 49]. However, astrocytes have been shown to internalize *α*Syn through a toll‐like receptor 4‐independent endocytosis pathway, with the *α*Syn localizing to lysosomes, suggesting that astrocytes are involved in the removal and degradation of these *α*Syn aggregates [50, 51, 52]. It is hypothesized that in the process of attempting to degrade the aggregates, some are stored, leading to the development of pathology [53, 54, 55]. The accumulation of *α*Syn in astrocytes can be affected by uptake, removal, transmission or other molecular mechanisms such as neuro‐inflammation [50, 54, 56, 57]. It is possible that the *α*Syn processing in astrocytes could alter the seeding capacity, in the form of increased cell‐to‐cell transfer between astrocytes or other cell‐types. Braak et al. reported that astrocytic *α*Syn inclusions were detected using antibodies targeting the 91–99 region of the non‐amyloid‐*β* component (i.e., a terminology used at the time of that publication) region, again, suggesting a different cellular processing of internalized *α*Syn in astrocytes of LBD [5]. Different carboxy terminal truncations of *α*Syn in different cell types in both MSA and LBD have also been recently reported, suggesting one of the factors underlying the cell type‐specific seeding differences [58].

More recently, Otero‐Jimenez et al. reported the potential progression patterns of cell type‐specific *α*Syn in PD using the Subtype and Stage Inference (SuStaIn) model and also found that morphologies of astrocytic‐αSyn may vary depending on the severity and stages of the disease, emphasizing the importance of cell type‐specific evaluation of *α*Syn [59]. Indeed, our study focused on how cell type‐specific *α*Syn pathology contributes to seeding differences in LBD irrespective of the clinical phenotype (i.e., PD, PDD, or DLB) and MSA. Evaluation of seeding differences between the clinical phenotypes associated with LBD, including stage‐related cytopathological differences, merits further studies.

Our study is subject to some limitations. One of the limitations is the accuracy of the proportion of αSyn cytopathology measured. The measurement of *α*Syn cytopathology was performed on a 4.5‐μm thick section of the TMA core. This is not an exact measurement of the cytopathology present throughout the depth of the 2‐mm core, but it represents the estimated cytopathological make‐up of each sample. Another limitation is that SAA studies should be complemented with further biochemical analysis. However, biochemical analysis such as protease digestion or visualization using western blot requires a larger amount of tissue. Obtaining a highly concentrated protein sample sufficient for biochemical analysis is difficult when extracting from a 2‐mm FFPE core by using the minimum number of protein‐altering factors in the process for the purpose of SAA. Our previous studies showed that proteins extracted from FFPE brain tissue preserve both disease‐specific and regional differences in seeding [26]. However, we cannot exclude the possibility of extracting a very low number of seeds in some samples, resulting in seeding that may not necessarily reflect cell type‐specific differences. To address this limitation, we used machine learning techniques to cluster based on important features while ignoring the less important details (i.e., seeding behaviors that may not reflect qualitative features), and found one cluster with samples that had low or negative seeding in LBD. Importantly, the aim of this study was not to correlate the amount of cell type‐specific *α*Syn with SAA parameters, but to explore whether the proportion of cell type‐specific *α*Syn contributes to different seeding. Finally, we cannot exclude the possibility that yet unidentified regional differences in the cellular microenvironment also contribute to the variability of seeding. However, our approach shows that those brain samples that correspond to predominant cell type‐specific *α*Syn, even if they represent different brain regions, seed in a similar fashion, supporting the notion that cytopathologies themselves also have an effect on the seeding.

In conclusion, our study highlights the effort to understand the potential cell type‐specific contribution to αSyn seeding in MSA and LBD. Although many different factors, either direct or indirect, can affect *α*Syn seeding, we observed different seeding behaviors in brain regions with distinct predominance of *α*Syn cytopathologies, providing insight on the potential relationship between the cytopathological make‐up and αSyn seeding behavior. Our study builds on the hypothesis that distinct conformations of misfolded *α*Syn result in different polymorphs or strains, each with distinct rates of cell‐to‐cell propagation and a preference for targeting specific cell types. We expand this concept by proposing that once internalized or produced by a particular cell type, the cellular processing of the misfolded *α*Syn may differ, leading to different seeding activities. Our results encourage further studies that evaluate cell type‐specific molecular processing (i.e., internalization mechanisms, selective vulnerability, inflammation, etc.) or cellular environment that work in concert for the pathogenesis of synucleinopathies [28, 60]. We highlight the potential influence of cell type‐specific *α*Syn on the seeding variability observed between cases and regions that will be relevant for basic researchers developing novel antibodies against cell‐specific *α*Syn and investigating the underlying cell type‐specific mechanisms of *α*Syn spreading.

## AUTHOR CONTRIBUTIONS

*Conceptualization*: Ain Kim, Gabor G. Kovacs. *Methodology*: Ain Kim, Ivan Martinez‐Valbuena, Shelley L. Forrest, Gabor G. Kovacs. *Validation*: Ain Kim, Gabor G. Kovacs. *Formal analysis and investigation*: Ain Kim, Gabor G. Kovacs. *Resources*: Krisztina Danics, Gabor G. Kovacs. *Data curation*: Ain Kim, Gabor G. Kovacs. *Writing—original draft preparation*: Ain Kim, Gabor G. Kovacs. *Writing—review and editing*: Ain Kim, Ivan Martinez‐Valbuena, Krisztina Danics, Shelley L. Forrest, Gabor G. Kovacs. *Visualization*: Ain Kim, Gabor G. Kovacs. *Supervision*: Gabor G. Kovacs. *Funding acquisition*: Gabor G. Kovacs.

## FUNDING INFORMATION

This project was funded by the Rossy Family Foundation, the MSA Coalition (grant number MSAC‐2022‐12‐003); the Edmond J. Safra Philanthropic Foundation; the Krembil Foundation, the Maybank Foundation, and the Canadian Foundation for Innovation (CFI) John R. Evans Leaders Fund (40480). Research reported in this publication was supported by the National Institutes of Health under award number 3200005870‐24‐112. Gabor G. Kovacs holds the Rossy Chair for PSP research. The funding bodies did not take part in the design of the study, in the collection, analysis, or interpretation of data, or in writing the manuscript.

## CONFLICT OF INTEREST STATEMENT

Gabor G. Kovacs holds a shared patent for the 5G4 synuclein antibody. Gabor G. Kovacs and Ivan Martinez‐Valbuena have a shared pending patent for diagnostic assays for movement disorders (18/537,455). Ain Kim, Krisztina Danics, and Shelley L. Forrest declare no competing interests.

## ETHICS STATEMENT

This study was approved by the University Health Network Research Ethics Board (Nr. 20‐5258) and the Regional and Institutional Committee of Science and Research Ethics, Semmelweis University (Nr. 34/2016).

## Supporting information


**Supplementary Figure 1.** Summarized schematic of the methods. Neuropathologically confirmed MSA (*n* = 10) and LBD cases (*n* = 15) have been selected. Specific areas in each brain region containing dominant neuronal‐ (i.e., neuronal cytoplasmic and neuritic), astrocytic‐ or oligodendrocytic‐*α*Syn was identified under the microscope. Using the outlined tissue section, the corresponding area was collected using a 2‐mm micro‐needle and each core was placed into the TMA mold. TMA cores were sectioned with the microtome, then each core was carefully removed for protein extraction. Following the optimized FFPE protein extraction, SAA was performed. The TMA sections were first immunohistochemically stained with the 5G4 antibody to confirm collection of the dominant cell type‐specific *α*Syn. Then the subsequent sections were double‐labeled using a cell marker (i.e., MAP2 for neurons, GLT‐1 for astrocytes and TPPP for oligodendrocytes) and 5G4 that labels the disease‐associated *α*Syn. Each double‐labeled TMA core was digitally scanned using the Nikon confocal microscope and HALO was used to quantify co‐localized cells. Manual counting was required for astrocytic‐*α*Syn. Seeding kinetics were plotted and parameters (i.e., AUC, lag phase, T50 and maximum ThT) were calculated by fitting the four‐parameter logistic model to the averaged fluorescence data using Python. Each cytopathology‐linked αSyn seeding profile and copathology scores of different regions and cases were then analyzed using machine learning algorithms and followed up with post‐hoc analysis to compare both pathologic and demographic features between clusters generated using k‐means clustering. Abbreviations: *α*Syn, *α*‐synuclein; LBD, Lewy body disease; MSA, multiple system atrophy; SAA, seed amplification assay; ThT, thioflavin T.
**Supplementary Figure 2**. Regions representing severity of tau and amyloid‐β scores. The severity of tau tangles in increasing severity are scored as follows: score 0 (A), score 1 (B), score 2 (C) and score 3 (D). The severity of amyloid‐β plaques in increasing pathological burden are scored as follows: score 0 (E), score 1 (F), score 2 (G) and score 3 (H).
**Supplementary Figure 3**. Inclusion criteria for quantifying GLT‐1+ astrocytes on double‐labeled TMA cores. GLT‐1+ cells that had either a visible nucleus or a GLT‐1+ morphology around a non‐visible nucleus with radiating processes (either few or many) were considered as 1 astrocyte. In some occasions, GLT‐1+ processes without visible nucleus or GLT‐1+ morphology around a non‐visible nucleus was considered as 1 astrocyte if the processes were radiating in a circular position and if these processes overlapped so that it seemed like 2 overlapping astrocytes, it was still quantified as 1 astrocyte as the exact quantity is unclear without a visible nucleus or a GLT‐1+ morphology around a non‐visible nucleus. All other morphologies, including astrocytic branches that overlapped over a large area, were excluded from the manual counting.
**Supplementary Figure 4**. A summary of the clustering results in MSA and LBD. In MSA, the highest αSyn seeding activity was observed in regions with predominance of oligodendrocytic‐*α*Syn (A). Although the proportion of oligodendrocytic‐*α*Syn was high in both clusters, seeding was lower when there was an increase in the neuronal involvement (i.e., higher proportion of neuronal cytoplasmic‐*α*Syn) (B). In LBD, the highest *α*Syn seeding activity is observed in regions that have a predominance of both neuronal cytoplasmic‐ and astrocytic‐*α*Syn, which are mostly regions that are affected in the later stages of the disease (C) while a lower seeding activity is observed in regions with a predominance of neuronal cytoplasmic‐*α*Syn, which are mostly regions that are affected in the early stages of the disease (D).

## Data Availability

The data generated and analyzed in this study are available from the corresponding author upon reasonable request.
