# Supplementary material for: Contribution of α‐synuclein cytopathologies to distinct seeding of misfolded α‐synuclein
Source: Brain Pathol. 2025 Jun 16;35(6):e70024. doi: 10.1111/bpa.70024 (PMC12488258; doi:10.1111/bpa.70024)
Supplement: Supplementary file 1 — Supplementary Figure 1. Summarized schematic of the methods. Neuropathologically confirmed MSA (n = 10) and LBD cases (n = 15) have been selected. Specific areas in each brain region containing dominant neuronal‐ (i.e., neuronal cytoplasmic and neuritic), astrocytic‐ or oligodendrocytic‐αSyn was identified under the microscope. Using the outlined tissue section, the corresponding area was collected using a 2‐mm micro‐needle and each core was placed into the TMA mold. TMA cores were sectioned with the microtome, then each core was carefully removed for protein extraction. Following the optimized FFPE protein extraction, SAA was performed. The TMA sections were first immunohistochemically stained with the 5G4 antibody to confirm collection of the dominant cell type‐specific αSyn. Then the subsequent sections were double‐labeled using a cell marker (i.e., MAP2 for neurons, GLT‐1 for astrocytes and TPPP for oligodendrocytes) and 5G4 that labels the disease‐associated αSyn. Each double‐labeled TMA core was digitally scanned using the Nikon confocal microscope and HALO was used to quantify co‐localized cells. Manual counting was required for astrocytic‐αSyn. Seeding kinetics were plotted and parameters (i.e., AUC, lag phase, T50 and maximum ThT) were calculated by fitting the four‐parameter logistic model to the averaged fluorescence data using Python. Each cytopathology‐linked αSyn seeding profile and copathology scores of different regions and cases were then analyzed using machine learning algorithms and followed up with post‐hoc analysis to compare both pathologic and demographic features between clusters generated using k‐means clustering. Abbreviations: αSyn, α‐synuclein; LBD, Lewy body disease; MSA, multiple system atrophy; SAA, seed amplification assay; ThT, thioflavin T. Supplementary Figure 2. Regions representing severity of tau and amyloid‐β scores. The severity of tau tangles in increasing severity are scored as follows: score 0 (A), score 1 (B), score 2 (C) [file BPA-35-e70024-s001.pdf]

Online Supplementary File

Contribution of α-Synuclein cytopathologies to distinct seeding of misfolded α-Synuclein

Ain Kim[1,2], Ivan Martinez-Valbuena[2,3], Krisztina Danics[5,6], Shelley L. Forrest[2,3], Gabor G. Kovacs[1,2,3,4,7]*

[1]Department of Laboratory Medicine & Pathobiology, University of Toronto, Toronto, ON, Canada

[2]Tanz Centre for Research in Neurodegenerative Disease, University of Toronto, Toronto, ON, Canada

[3]Krembil Brain Institute, University Health Network, Toronto, ON, Canada

[4]Edmond J. Safra Program in Parkinson's Disease and the Morton and Gloria Shulman Movement Disorders Clinic, Toronto Western Hospital, Toronto, ON M5T 2S6, Canada

[5]Department of Forensic and Insurance Medicine, Semmelweis University, Budapest, 1085, Hungary

[6]Neuropathology and Prion Disease Reference Center, Department of Forensic and Insurance Medicine, Semmelweis University, Budapest, 1091, Hungary

[7]Laboratory Medicine Program, University Health Network, Toronto, ON M5G 2C4, Canada

Correspondence: Gabor G. Kovacs. University of Toronto, Tanz Centre for Research in Neurodegenerative Disease (CRND), Krembil Discovery Tower, 60 Leonard Ave Toronto, ON, M5T 0S8, Canada; Tel: +1 (416) 507-6858

Email: gabor.kovacs@uhn.ca

SUPPLEMENTARY MATERIAL

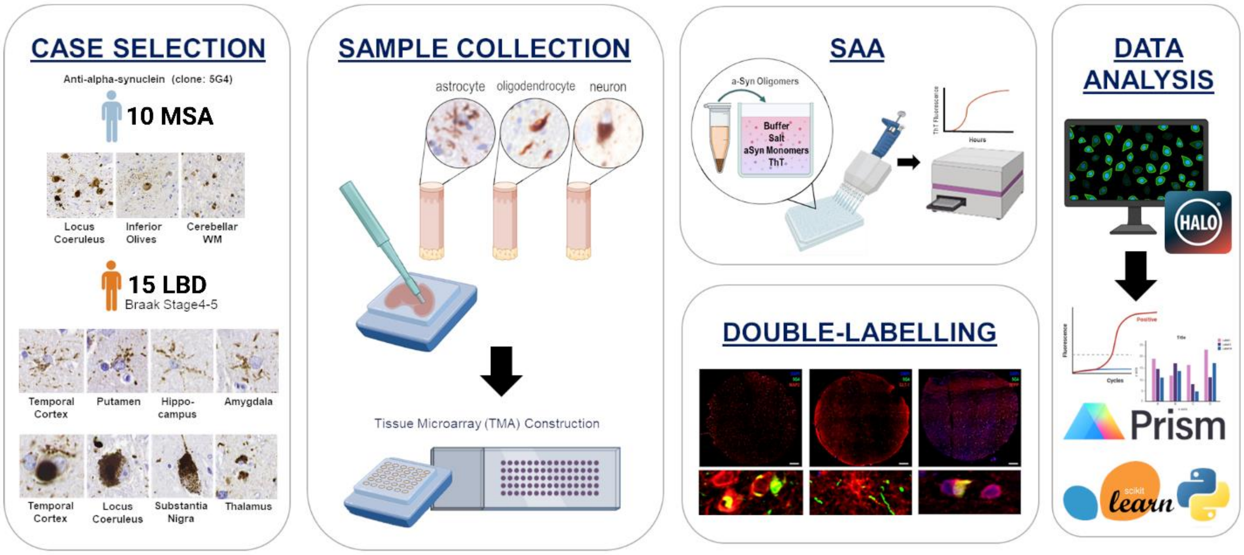

Supplementary Figure 1. Summarized schematic of the methods. Neuropathologically confirmed MSA (n = 10) and LBD cases (n = 15) have been selected. Specific areas in each brain region containing dominant neuronal- (i.e., neuronal cytoplasmic and neuritic), astrocytic- or oligodendrocytic-αSyn was identified under the microscope. Using the outlined tissue section, the corresponding area was collected using a 2-mm micro-needle and each core was placed into the TMA mold. TMA cores were sectioned with the microtome, then each core was carefully removed for protein extraction. Following the optimized FFPE protein extraction, SAA was performed. The TMA sections were first immunohistochemically stained with the 5G4 antibody to confirm collection of the dominant cell type-specific αSyn. Then the subsequent sections were double-labeled using a cell marker (i.e., MAP2 for neurons, GLT-1 for astrocytes and TPPP for oligodendrocytes) and 5G4 that labels the disease-associated αSyn. Each double-labeled TMA core was digitally scanned using the Nikon confocal microscope and HALO was used to quantify co-localized cells. Manual counting was required for astrocytic-αSyn. Seeding kinetics were plotted and parameters (i.e., AUC, lag phase, T50 and maximum ThT) were calculated by fitting the four-parameter logistic model to the averaged fluorescence data using Python. Each cytopathology-linked αSyn seeding profile and co-pathology scores of different regions and cases were then analyzed using machine learning algorithms and followed up with post-hoc analysis to compare both pathologic and demographic features between clusters generated using k-means clustering. Abbreviations: αSyn, α-synuclein; LBD, Lewy body disease; MSA, multiple system atrophy; SAA, seed amplification assay; ThT, thioflavin T.

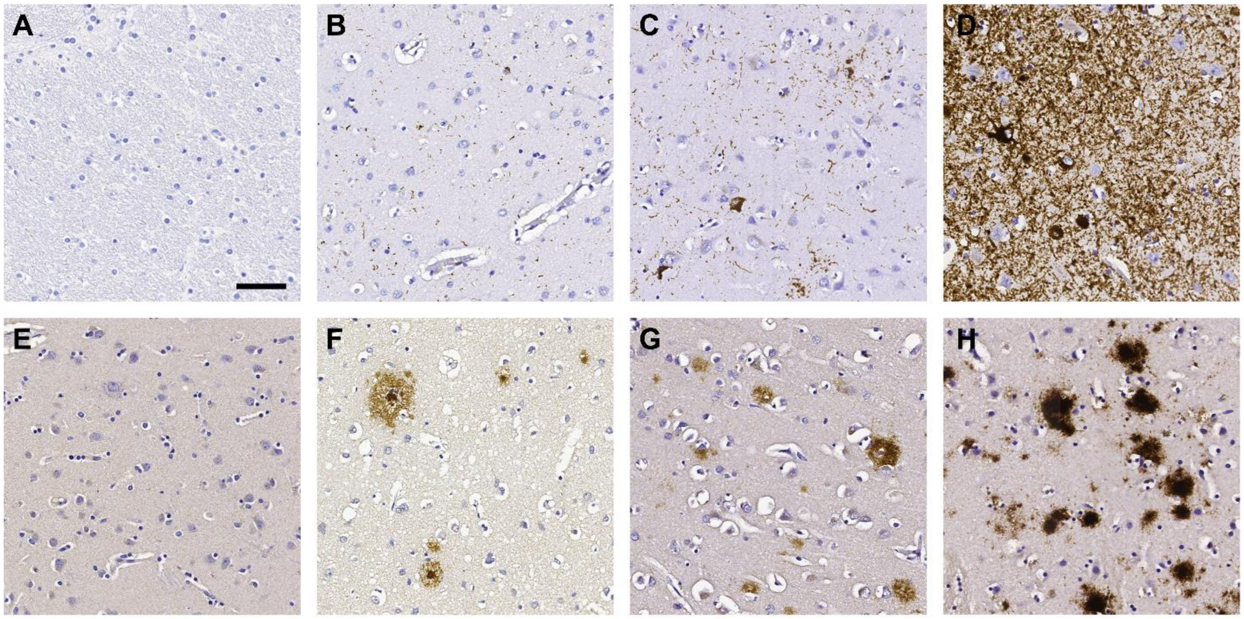

Supplementary Figure 2. Regions representing severity of tau and amyloid-β scores. The severity of tau tangles in increasing severity are scored as follows: score 0 (A), score 1 (B), score 2 (C) and score 3 (D). The severity of amyloid-β plaques in increasing pathological burden are scored as follows: score 0 (E), score 1 (F), score 2 (G) and score 3 (H). Scale bar in (A) represent 50 μm and applies to all images.

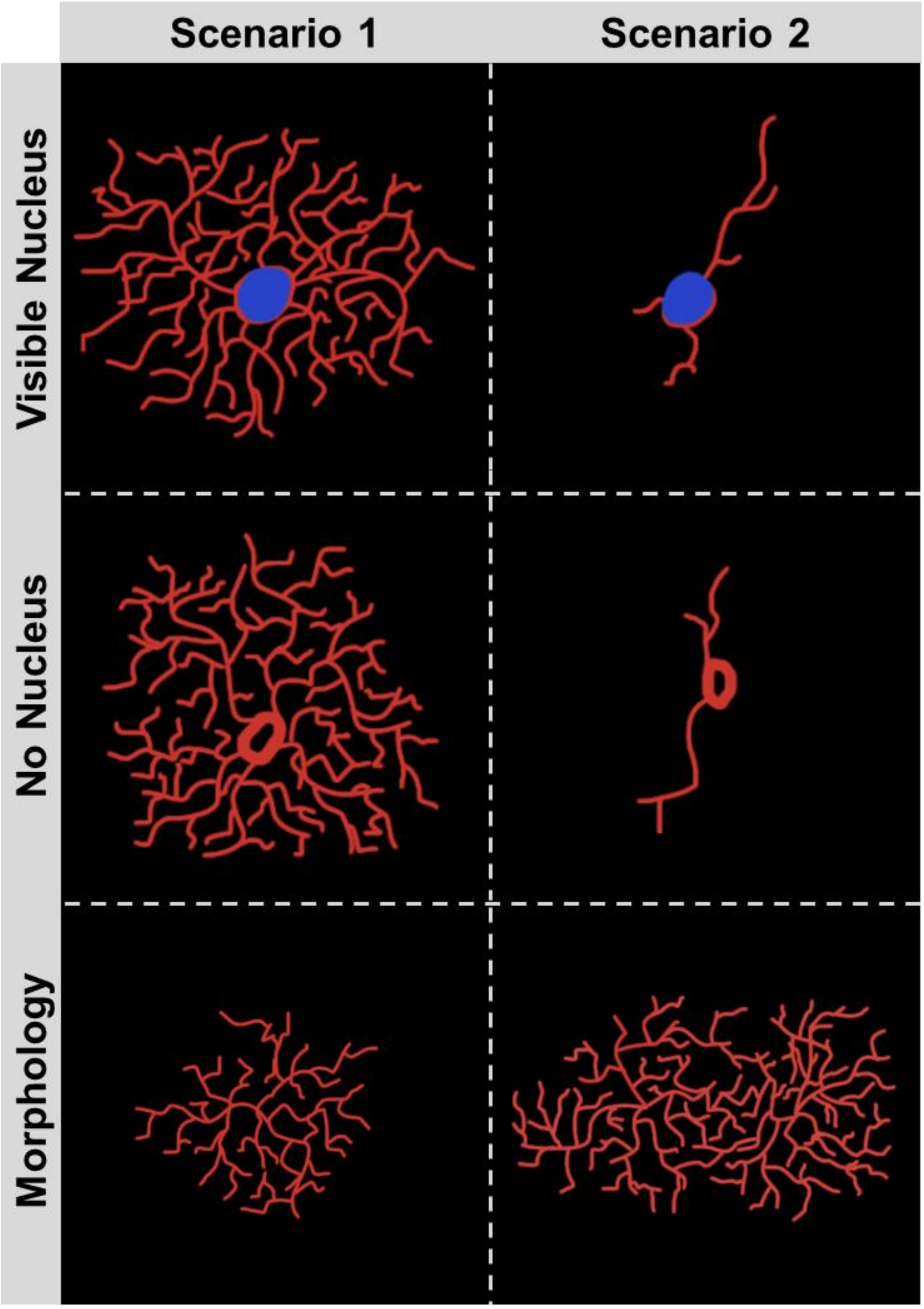

Supplementary Figure 3. Inclusion criteria for quantifying GLT-1+ astrocytes on double-labeled TMA cores. GLT-1+ cells that had either a visible nucleus or a GLT-1+ morphology around a non-visible nucleus with radiating processes (either few or many) were considered as 1 astrocyte. In some occasions, GLT-1+ processes without visible nucleus or GLT-1+ morphology around a non-visible nucleus was considered as 1 astrocyte if the processes were radiating in a circular position. If these processes overlapped so that it seemed like 2 overlapping astrocytes, it was still quantified as 1 astrocyte as the exact quantity is unclear without a visible nucleus or a GLT-1+ morphology around a non-visible nucleus. All other morphologies, including astrocytic branches that overlapped over a large area, were excluded from the manual counting.

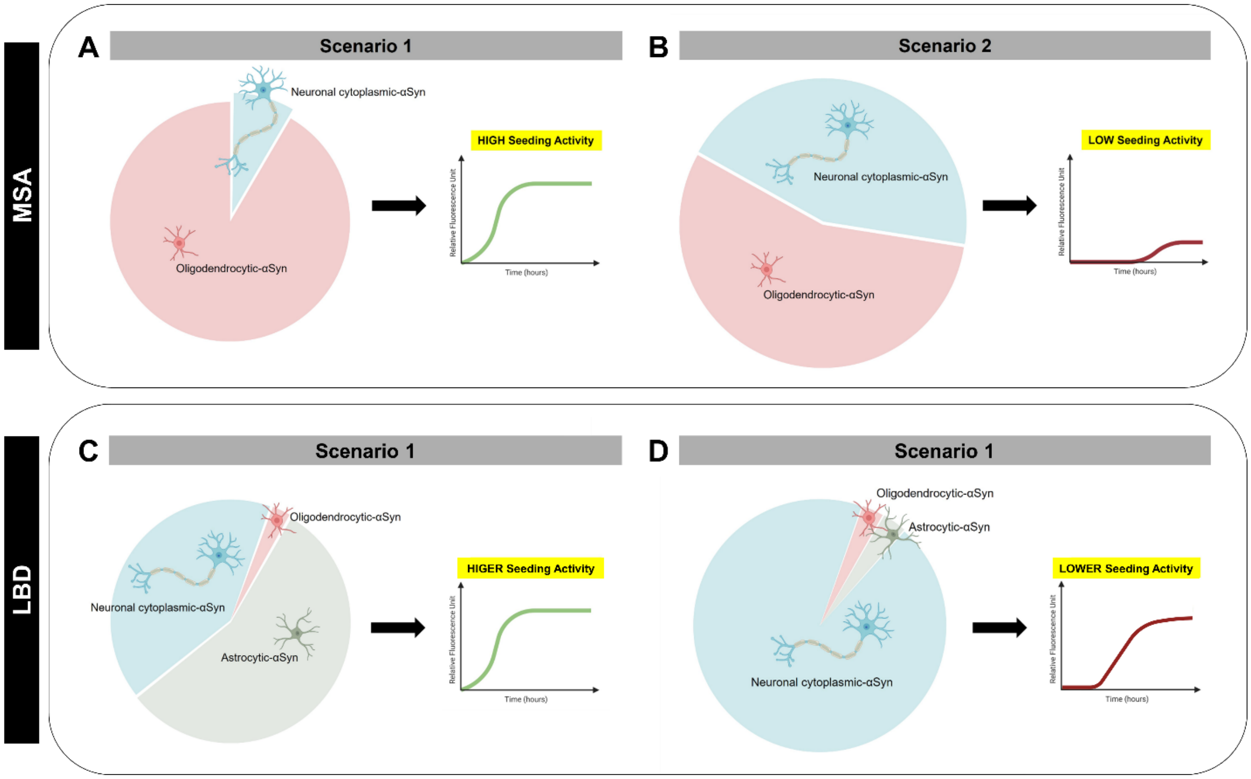

Supplementary Figure 4. A summary of the clustering results in MSA and LBD. In MSA, the highest αSyn seeding activity was observed in regions with predominance of oligodendrocytic-αSyn (A). Although the proportion of oligodendrocytic-αSyn was high in both clusters, seeding was lower when there was an increase in the neuronal involvement (i.e., higher proportion of neuronal cytoplasmic-αSyn) (B). In LBD, the highest αSyn seeding activity is observed in regions that have a predominance of both neuronal cytoplasmic- and astrocytic-αSyn, which are mostly regions that are affected in the later stages of the disease (C) while a lower seeding activity is observed in regions with a predominance of neuronal cytoplasmic-αSyn, which are mostly regions that are affected in the early stages of the disease (D).
